# Galeterone and its analogs inhibit Mnk-eIF4E axis, synergize with gemcitabine, impede pancreatic cancer cell migration, invasion and proliferation and inhibit tumor growth in mice

**DOI:** 10.18632/oncotarget.14154

**Published:** 2016-12-24

**Authors:** Andrew K. Kwegyir-Afful, Francis N. Murigi, Puranik Purushottamachar, Vidya P. Ramamurthy, Marlena S. Martin, Vincent C.O. Njar

**Affiliations:** ^1^ Department of Pharmacology, University of Maryland School of Medicine, Baltimore, MD 21201-1559, USA; ^2^ Center for Biomolecular Therapeutics, University of Maryland School of Medicine, Baltimore, MD 21201-1559, USA; ^3^ Marlene and Stewart Greenebaum Comprehensive Cancer Center, University of Maryland School of Medicine, Baltimore, MD 21201-1559, USA; ^4^ Current Address: Bernard J. Dunn School of Pharmacy, Shenandoah University, Ashburn, VA 20147, USA

**Keywords:** pancreatic cancer resistance, galeterone (gal) and its analogs

## Abstract

Survival rate for pancreatic cancer (pancreatic ductal adenocarcinoma, PDAC) is poor, with about 80% of patients presenting with the metastatic disease. Gemcitabine, the standard chemotherapeutic agent for locally advanced and metastatic PDAC has limited efficacy, attributed to innate/acquired resistance and activation of pro-survival pathways. The Mnk1/2-eIF4E and NF-κB signaling pathways are implicated in PDAC disease progression/metastasis and also associated with gemcitabine-induced resistance in PDAC. Galeterone (gal), a multi-target, agent in phase III clinical development for prostate cancer has also shown effects on the aforementioned pathways. We show for the first time, that gal/analogs (VNPT55, VNPP414 and VNPP433-3β) profoundly inhibited cell viability of gemcitabine-naive/resistance PDAC cell lines and strongly synergized with gemcitabine in gemcitabine-resistant PDAC cells. In addition, to inducing G1 cell cycle arrest, gal/analogs induced caspase 3-mediated cell-death of PDAC cells. Gal/analogs caused profound downregulation of Mnk1/2, peIF4E and NF-κB (p-p65), metastatic inducing factors (N-cadherin, MMP-1/-2/-9, Slug, Snail and CXCR4) and putative stem cell factors, (β-Catenin, Nanog, BMI-1 and Oct-4). Gal/analog also depleted EZH2 and upregulated E-Cadherin. These effects resulted in significant inhibition of PDAC cell migration, invasion and proliferation. Importantly, we also observed strong MiaPaca-2 tumor xenograft growth inhibition (61% to 92%). Collectively, these promising findings strongly support further development of gal/analogs as novel therapeutics for PDAC.

## INTRODUCTION

Pancreatic cancer (pancreatic ductal adenocarcinoma, PDAC) is a highly aggressive epithelial cancer with a reported 5-year survival rate of ∼5% [1]. Only 20% of pancreatic cancer patients are eligible for surgical resection, and the metastatic disease frequently develops even after surgery, while current chemo- and radio-therapies are largely ineffective [2]. The K-RAS oncogene is most frequently (90%) mutated in pancreatic tumors and a key driver of the disease [3]. Mutant K-RAS oncogene constitutively activates the MAPK pathway as well as the PI3K/Akt/mTOR pathway, which are known to promote growth and development of PDAC [4, 5]. These two signaling pathways converge downstream at the eukaryotic translational initiation complex, eIF4F, which mediates cap-dependent mRNA translational initiation apparatus critical for eukaryotic protein synthesis [6–8].

Phosphorylation of eIF4E at serine 209 by the MAPK interacting kinases Mnk1/2 [9, 10] is a rate-limiting step in the formation of the eIF4F complex preceding mRNA translation. Mnk1/2 induced eIF4E phosphorylation is a vital oncogenic occurrence that enhances selective translation of oncogenic mRNAs, implicated in cell growth (c-Myc and cyclin D1), cell survival and evasion of apoptosis (Mcl-1 and Bcl-2), metastasis (MMP-9) or angiogenesis (VEGF and FGF2) [11, 12]. Inhibitors targeting the Mnk1/2-eIF4E axis are actively under investigation for the treatment of several cancers [13].

Advanced and or metastatic pancreatic cancer patients who have previously received chemotherapy with unresectable tumors are normally treated with gemcitabine, which however, shows very limited efficacy [14]. To improve PDAC therapy, clinical trials combining drugs with gemcitabine to sensitize pancreatic tumors have been conducted [15]. In a recent study, screening of a cohort of PDAC patients by immunocytochemistry showed that eIF4E phosphorylation correlated with early onset of the disease, disease grade, and deplorable prognosis [16]. This study also demonstrated that gemcitabine triggers a pro-survival response in PDAC cells through activation of Mnk2-eIF4E pathway [16]. Gemcitabine reportedly also increases activation of the NF-κB pathway which plays a vital role in gemcitabine resistance in PDAC [17, 18]. Several preclinical studies have evaluated the significance of inhibiting Mnk1/2-eIF4E axis to prevent the growth of PDAC cells and tumors *in vitro* and *in vivo* [16, 19]. Other studies have also shown with organoid cultures and co-culturing PDAC cells with matrix fibroblast, the significance of the mRNA translation machinery, it's up-regulation and pivotal role in tumor initiation and growth [20, 21]. These studies remarkably delineated the mechanisms of tumor growth inhibition resulting from Mnk1/2-eIF4E axis antagonism.

Our group has been developing small molecule inhibitors for the treatment of metastatic castration resistant prostate cancer [22]. With increasing evidence of the significance of the translation machinery in cancer disease progression and metastasis, we evaluated the effects of our lead compounds on the Mnk1/2-eIF4E cap-dependent mRNA translation complex. Our previous published work suggested that gal exhibited effects on the translation machinery by exerting depletion effects on cyclin D1 which is tightly regulated by the cap dependent translation machinery and also downregulating eIF2α phosphorylation [23]. Our recent study with gal and VNPT55 on prostate cancer cell migration, reveal the extensive impact of downregulating Mnk1/2-eIF4E on EMT and putative stem cell factors [24]. This extensive study revealed that galeterone and its analog, VNPT55 markedly depleted protein expression of Mnk1/2 and downregulated phosphorylation of eIF4E. Silencing Mnk1 genomically also resulted in the downregulation of several oncogenic biomarkers implicated in drug-resistance, EMT and stem cell renewal [24]. Gal has been studied in over 250 patients with no detectable host toxicity [22, 25]. Gal antagonizes androgen receptor (AR) signaling [26], induces apoptosis [27] and endoplasmic reticulum stress response (ERSR) [23]. Gal also inhibits the growth of AR negative prostate cancer (PC) cells [23]. Current studies revealed that gal/analogs deplete protein expression of Mnk1/2 which results in downregulation of eIF4E phosphorylation in prostate [24]. This, in addition to reports on the expression of AR and the potential use of AR blocking agents in PDAC cells [28] prompted us to evaluate the efficacy of gal and its novel analogs in PDAC.

Unlike prostate cancer cell lines, very few PDAC cells express relatively lower levels of AR protein, whereas others lack any detectable AR expression [29]. Since our current studies have shown strong effects of gal/analogs on the Mnk1/2-eIF4E axis and the latter is implicated in oncogenesis and gemcitabine resistance in pancreatic cancer [30], we hypothesize that gal/analogs’ effects on Mnk1/2 could greatly influence their activity in PDAC cells lines and xenograft tumors. Our *in vitro* studies utilized a number cell lines acquired from primary localized tumors, ascites, metastatic lesions and drug-resistant cells, which would suggest that although ‘drug’-activity may vary in different cell lines expressing myriad diverse mutations and overexpressed oncogenes, gal/analogs exhibit similar and comparable potency/activity in most PDAC cells lines.

**Figure 1:**
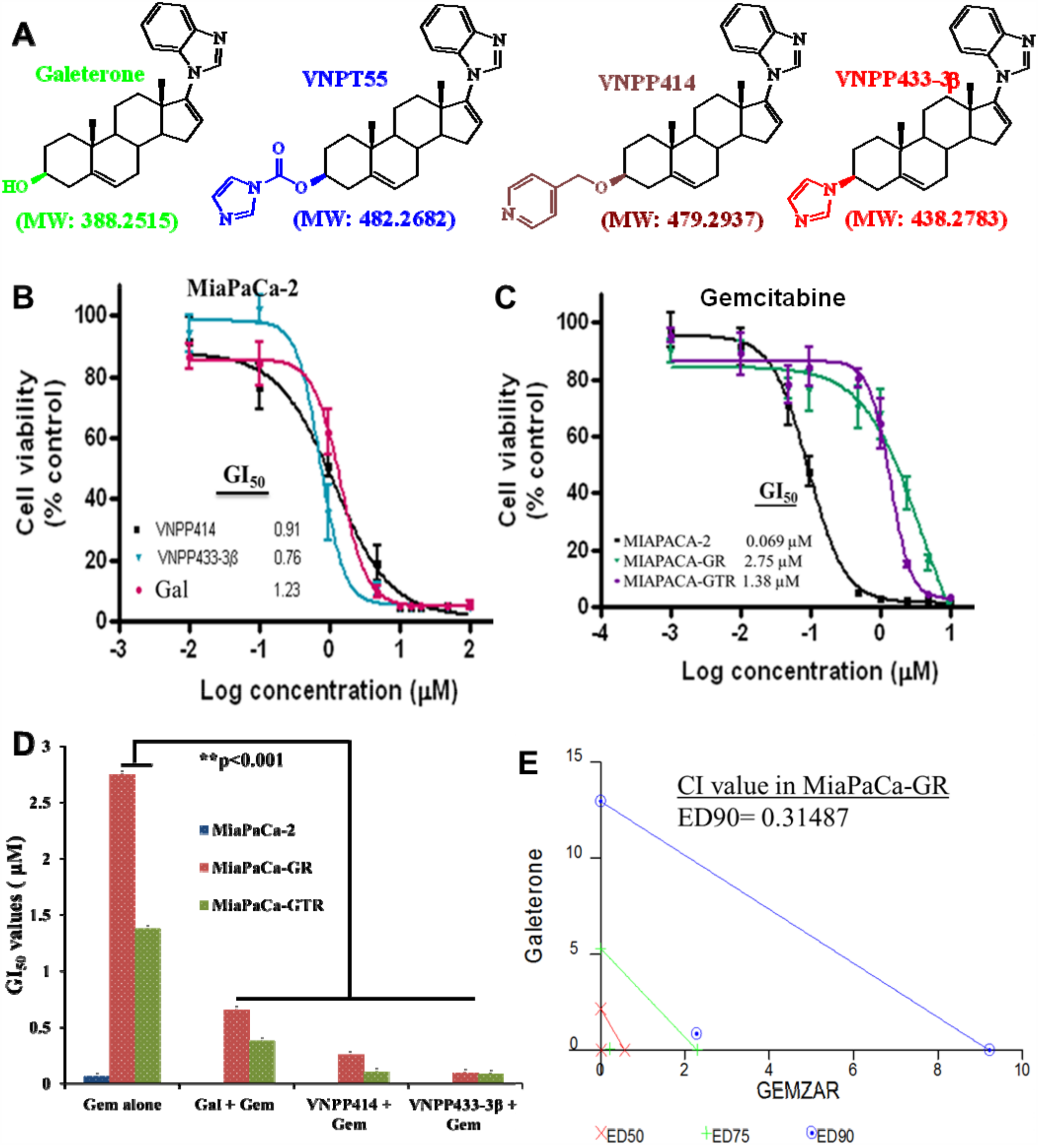
Anti-proliferative activities of ARDAs and gemcitabine in PDAC cells **A**. Structures of Androgen Receptor Degrading Agents (ARDA). **B**. Gal and analogs decrease cell viability of MiaPaCa-2 cells utilizing MTT cell viability assays. **C**. MTT cell viability assays in drug-naive and drug-resistant (MiaPaCa-2, MiaPaCa-GR and MiaPaCa-GTR) show a decrease in gemcitabine activity in drug-resistant cells **D**. Gal and analogs potentiate effects of gemcitabine and strikingly decrease GI_50_values in gemcitabine-resistant PDAC cells (**p < 0.001). PDAC cells were first treated with gal and analogs, then with gemcitabine. **E**. Gal and gemcitabine synergistically inhibit cell viability of gemcitabine-resistant PDAC cells. Cell viability assays were conducted for gal and gemcitabine individually and GI_50_ values calculated. Both compounds were combined at a constant ratio and utilized in cell viability assays. Fractional effects of single agents and in combination were calculated and analyzed by the calcusyn software to compute the combination indices (CI) at ED50, ED75 and ED90. CI value at ED90 reveal strong synergy between the two compounds in MiaPaCa-GR cells.

Pancreatic cancer cell lines that are utilized in preclinical studies harbor a varying genetic backgrounds. Thus, our initial study was to determine whether the multiple target effects of gal and its analogs would enhance their anticancer activity in PDAC cells and xenograft. In the present study, we show that, gal and its analogs (Figure 1A) significantly inhibited cell viability of both gemcitabine-naïve/resistant PDAC cells and strongly synergized with gemcitabine in gemcitabine-resistant cells. We detected remarkable depletion effect on epithelial-mesenchymal-transition (EMT) and putative stem cancer cell markers. In addition, gal and its analogs markedly downregulated NF-κB (p65) phosphorylation in both cells acquired from localized tumors (MiaPaCa-2) and metastatic lesions (S2-013). We also observed significant anti-migratory and anti-invasive activities in gemcitabine-naïve/resistant PDAC cells. We provide evidence for the first time to suggest that gal/analogs possess excellent antitumor activities against MiaPaCa-2 PDAC xenografts in mice. Protein expression analysis show profound induction of apoptosis and downregulation of Mnk1/2 and peIF4E *in vitro* and *in vivo*. A preliminary account of part of this work has recently been reported [30].

## RESULTS

### Gal/analogs profoundly decrease PDAC cell viability and synergize with gemcitabine in drug-resistant PDAC cells

Rigorous cell viability assays in drug-naïve (MiaPaCa-2) (Figure 1B), metastatic (S2-013) and gemcitabine-resistant PDAC cells (MiaPaCa-GR: *resistant to 200 nM gemcitabine* & MiaPaCa-GTR: *resistant to gemcitabine (200 nM) and Erlotinib (2 μM)*) (Figure 1C and Table 1), demonstrated that gal and analogs possess exceptional anti-proliferative activities and decreased PDAC cell viability, with GI_50_ values as low as 0.15 to 7.08 μM (Figure 1C and Table 1). Cell viability analysis with gemcitabine in drug-naïve and drug-resistant PDAC cells show that GI_50_ values of gemcitabine increased approximately 20 and 40-fold in MiaPaCa-GTR and MiaPaCa-GR cells, respectively (Figure 1C and Table 2).

**Table 1:** GI_50_ values^a^ of gal/analogs in gemcitabine-naive and gemcitabine-resistant PDAC cells

	GI_50_ Values (μM)		
Cell Lines	Galeterone	VNPP414	VNPP433-3β
MiaPaCa-2	1.23 ± 0.15	0.91 ± 0.03	0.76 ± 0.03
S2-013	0.49 ± 0.001	0.15 ± 0.001	0.46 ± 0.002
MiaPaCa-GR	7.08 ± 0.13	2.45 ± 0.11	1.23 ± 0.02
MiaPaCa-GTR	2.19 ± 0.03	1.48 ± 0.02	0.52 ± 0.003

Cells were seeded at 2500 cells/well in a 96-well plate and treated with serially diluted compounds for 72 h. Media was replenished with compounds for an additional 96 h. Cell viability was determined using MTT reagent and GI_50_ values computed by graphpad prism 4. Gal and analogs exhibit antiproliferative activities at low micromolar concentrations compared to DMSO-treated controls.

^a^GI_50_ values: Concentration of agent that inhibits cell proliferation by 50%.

**Table 2:** GI_50_ values^a^ of gemcitabine alone and in combination with gal/analogs in PDAC cells

	GI_50_ Values (μM)			
Cell Lines	Gem^b^ alone	Gal + Gem	VNPP414 + Gem	VNPP433-3β + Gem
MiaPaCa-2	0.069 ± 0.03			
MiaPaCa-GR	2.75 ± 0.11	0.66 ± 0.001	0.26 ± 0.001	0.1 ± 0.001
MiaPaCa-GTR	1.38 ± 0.01	0.38 ± 0.002	0.11 ± 0.003	0.09 ± 0.001

Sequential treatment with gal/analogs and gemcitabine in MTT cell viability assays shows a significant decrease in GI_50_ values in gemcitabine-resistant and gemcitabine/erlotinib-resistant cells compared to gemcitabine alone.

^a^GI_50_ values: Concentration of agent that inhibits cell proliferation by 50%.

^b^Gem: Gemcitabine

We also analyzed whether gal and analogs could sensitize gemcitabine-resistant cells and potentiate gemcitabine's activity in these cells. Hence, PDAC cells were sequentially treated with gal/analogs then gemcitabine. Analysis of GI_50_ values of consecutive treatment compared to gemcitabine alone (Figure 1C) show significantly decreased GI_50_ values (Figure 1D and Table 2). Average fold decrease in GI_50_ values observed in gemcitabine-resistant PDAC cells ranged from 3.6- to 27.5-fold in gemcitabine-resistant and gemcitabine /erlotinib-resistant PDAC cells.

Furthermore, we determined whether combining gal/analogs with gemcitabine at their respective GI_50_ values, would result in synergistic anti-proliferative activities. We utilized Chou and Talalay’s’ combination-index (CI) and isobologram approaches derived from the median effect principle [31]. Results from viability assays were used to determine the fraction affected (Fa) of cells by compounds. Doses of the different compounds resulting in 50% fraction affected of cell kill were selected and combined at a fixed ratio, data from combination treatment were analyzed with the calcusyn software (BioSoft, version 2.0). It was interesting to observe that, gal-gemcitabine combination in gemcitabine-resistant PDAC cells, resulted in very low CI values, indicating strong synergy, as represented by the isobologram in Figure 1E and CI values in Table 3. In gemcitabine-erlotinib resistant PDAC cells, VNPP433-3β in combination with gemcitabine even at ED90 exhibited strong synergy (Table 3) in combination with gemcitabine. These data suggests that gal/analogs could potentially be combined with gemcitabine in gemcitabine-resistant PDAC to elicit superior efficacy than gemcitabine alone.

**Table 3:** Combination Indices (CI values) for gal/analogs and gemcitabine combination studies in gemcitabine-resistant PDAC cells

Cell Lines	Combination @ ^a^GI_50_	CI values at		
		^b^ED50	^b^ED75	^b^ED90
MiaPaCa-GR	Galeterone + Gem	0.04459	0.31167	0.31487
MiaPaCa-GTR	VNPP433-3β + Gem	0.03321	0.12246	0.45757

CI values indicate synergy between gal/VNPP433-3β and gemcitabine in gemcitabine-resistant and gemcitabine-erlotinib-resistant PDAC cells (*MiaPaCa-GR and MiaPaCa-GTR*). Cell viability assays were conducted for gal and gemcitabine individually and GI_50_ values calculated. Compounds were subsequently combined at their GI_50_ (constant ratio). Fractional effects of single agents and in combination were calculated and analyzed by calcusyn software to compute the combination indices (CI) at ED50, ED75 and ED90. (CI<1-synergy, CI=1-additive and CI>1-antagonism. Observed CI values indicates strong synergy between gal/analogs and gemcitabine in the resistant cells.

^a^GI_50_: Concentration of agent that inhibits cell proliferation by 50%.

^b^ED50, ED75, ED90: Effective Dose at 50, 75 and 90% inhibitions

### Gal and analogs induce G1 cell cycle arrest and apoptosis in PDAC cells

**Figure 2:**
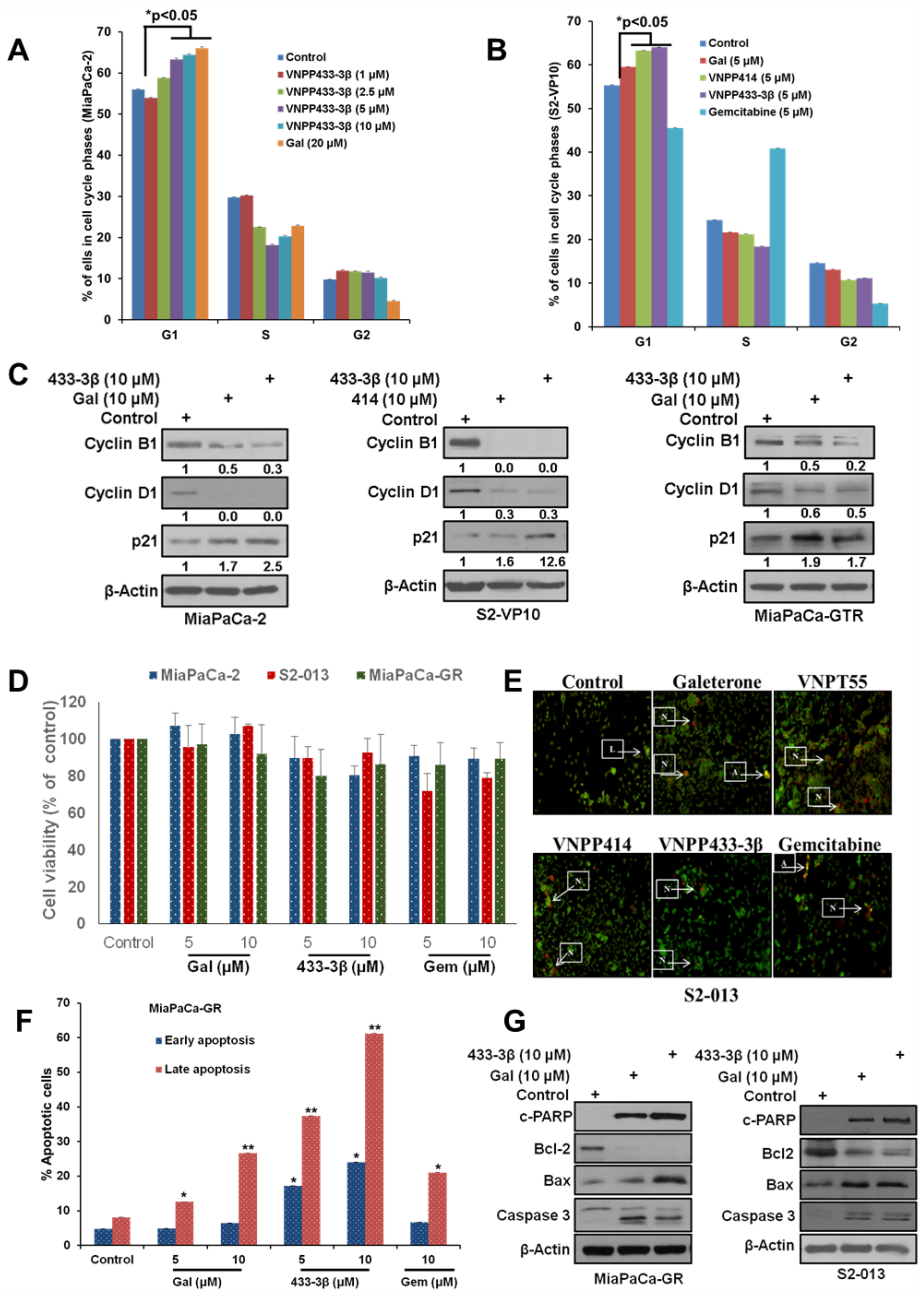
Gal/analogs and gemcitabine cause cell cycle arrest and induce apoptosis in PDAC cells **A**. Gal and VNPP433-3B, induce G1 cell cycle arrest in MiaPaCa-2 cells. **B**. Gal and analogs cause accumulation of cells in the G1 phase of cell cycle in metastatic PDAC cells (S2-VP10). **C**. Gal and analogs deplete cell cycle regulators (cyclin B1 and cyclin D1) and upregulate p21 in MiaPaCa-2, S2-VP10 and MiaPaCa-GTR cells. **D**. Cell viability assays indicate that concentrations utilized (5 and 10 μM), over a 24 h time point did not compromise cell viability significantly. **E**. Gal/analogs and gemcitabine induce apoptosis in S2-013 cells *in vitro*, using the acridine orange/ethidium bromide assay to determine loss of cell membrane integrity. Cells were treated at 2.5 μM for 72 h. Arrows next to L indicate live cells; arrows pointing to A indicate apoptotic cells; and arrows pointing N indicate necrotic cells **F**. Gal/VNPP433-3β and gemcitabine (5 and 10 μM) were compared in their ability to induce apoptosis, analyzed by flow cytometry. Cells treated with compounds for 24 h were stained with annexin v and propidium iodide (PI). Early and late apoptotic cells were analyzed by FACS. All compounds show significant apoptotic induction (*p < 0.05, **p < 0.001). **G**. Gal and analogs enhanced depletion of Bcl-2 and increased expression of cleaved PARP, caspase 3 and Bax in gemcitabine-naive/resistant PDAC cells.

To determine whether inhibition of cell cycle progression and apoptotic induction contributed to decrease in cell viability, induced by gal and analogs, we analyzed their effects on cell distribution at the different phases of the cell cycle at varying doses. In MiaPaCa-2 cells, treatment with gal or VNPP433-3β, resulted in dose-dependent increased accumulation of cells in the G1-phase, with a significantly noticeable reduction in S-phase (Figure 2A). In the metastatic cell line S2-VP10, contrary to gemcitabine where we observe a progression to S-phase, gal, VNPP414 or VNPP433-3β (5 μM, each), significantly induced G1 cell cycle arrest (Figure 2B). We next analyzed the effects of the compounds on cell cycle regulators (cyclin B1, cyclin D1 and p21), in MiaPaCa-2, MiaPaCa-GTR and S2-VP10 cells. We observed that the lead compounds depleted protein expression of cyclin B1, cyclin D1 and caused an upregulation of p21 (Figure 2C and 2D). These effects partly suggest a molecular explanation for the accumulation of cells in G1-phase (Figure 2A).

One of the most desirable properties of anti-cancer agents is the ability to induce apoptosis (cytotoxicity). Some compounds are known to be cytostatic [32, 33], and such properties have been implicated in their failure as therapeutic agents in the clinic [34]. Our previous studies showed that gal and VNPT55 induced apoptosis via up-regulation of Bax, cytochrome c release and activation of caspases in prostate cancer cells [27]. In our previous studies, we show that doses used in our 24 h - 72 h studies did not compromise cell viability or cell numbers significantly. In this study a 24 h our cell viability assay with gal, VNPP433-3β and gemcitabine (5 and 10 μM) also reproduced similar results, with no significant decrease in cell viability or changes in cell numbers compared to control (Figure 2D). We then utilized these doses to evaluate apoptotic induction using acridine orange/ethidium bromide and annexin-v (AO/EtBr) and propidium iodide (PI) stain assays to detect both early and late apoptosis. Our results show that, similar to gemcitabine's activity, all four lead compounds, at 2.5 μM (AO/EtBr) and 5 - 10 μM (Annexin v/PI), significantly induced apoptosis in S2-013 and MiaPaCa-GR cells, respectively (Figure 2E and 2F). We also observed that, gal/VNPP433-3β, at 10 μM, were remarkably superior to gemcitabine in MiaPaCa-GR cells (Figure 2F). Critical molecular regulators of apoptosis such as anti-apoptotic protein (Bcl-2), pro-apoptotic marker (Bax), caspase 3 and PARP cleavage, were analyzed to validate apoptotic induction observed in Figure 2E and 2F. We observed that, gal and VNPP433-3β profoundly depleted protein expression of Bcl-2, upregulated Bax protein expression and caused caspase 3 and PARP cleavage in both gemcitabine-naïve and resistant PDAC cells (Figure 2G).

### Gal and analogs target Mnk1/2-eIF4E axis in PDAC cells

**Figure 3:**
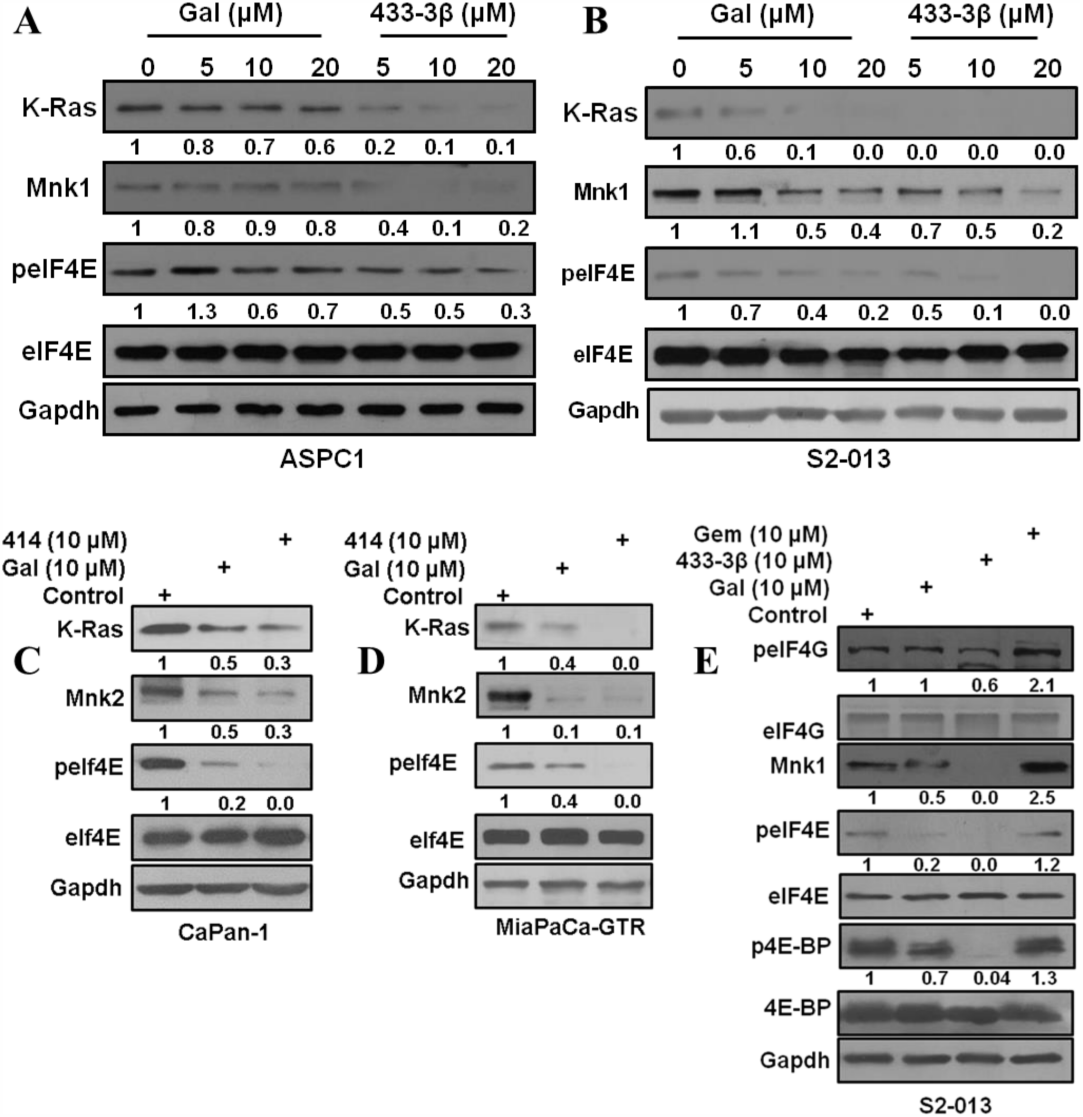
Gal and analogs deplete Mnk1/2 protein expression and downregulate eIF4E phosphorylation **A** and **B**. ASPC1 and S2-013 cells treated with gal and VNPP433-3β show a dose dependent decrease in K-Ras, Mnk1 and peIF4E. **C** and **D**. CaPan-1 and MiaPaCa-GTR cells treated with gal and VNPP414 at 10 μM also show decrease in Ras, Mnk2 and peIF4E protein expression. **E**. S2-013 cells treated with gal/VNPP433-3β/gemcitabine was analyzed for phosphorylation levels of eIF4G, eIF4E and 4E-BP. Protein expression analysis reveal an enhanced expression of Mnk1 by gemcitabine treatment.

Recent studies on further molecular characterization of gal/analogs, revealed profound inhibitory effects on the eukaryotic cap-dependent translation machinery (Mnk1/2-eIF4E pathway) [24]. Considering the role that Mnk-eIF4E pathway plays in cancer progression and the fact that Mnk2-eIF4E is constitutively activated during gemcitabine resistance [16], we evaluated the potential of gal/analogs to abrogate Mnk-eIF4E activation in PDAC as a plausible mechanism for their efficacy in gemcitabine-resistant cells. Gal and VNPP433-3β depleted K-Ras and Mnk1 protein expression dose-dependently with a concomitant down-regulation of peIF4E, but with no effects on total eIF4E in ASPC1 and S2-013 cells (Figure 3A and 3B). In addition, treatment of CaPan1 and MiaPaCa-GTR cells with 10 μM of gal/VNPP414 also caused depletion of Mnk2 and K-Ras protein expression and down-regulated peIF4E (Figure 3C and 3D).

A brief analysis of effects on protein expression of components of the cap-dependent translation machinery revealed that gal and VNPP433-3β downregulated phosphorylation of both 4E-BP1 and eIF4G. In contrast, gemcitabine treatment caused upregulation of eIF4G and 4E-BP1 phosphorylation in addition to an increase in Mnk1 protein expression (Figure 3E).

### Gal and analogs antagonize the NF-κB pathway and decrease EMT markers in PDAC cells

**Figure 4:**
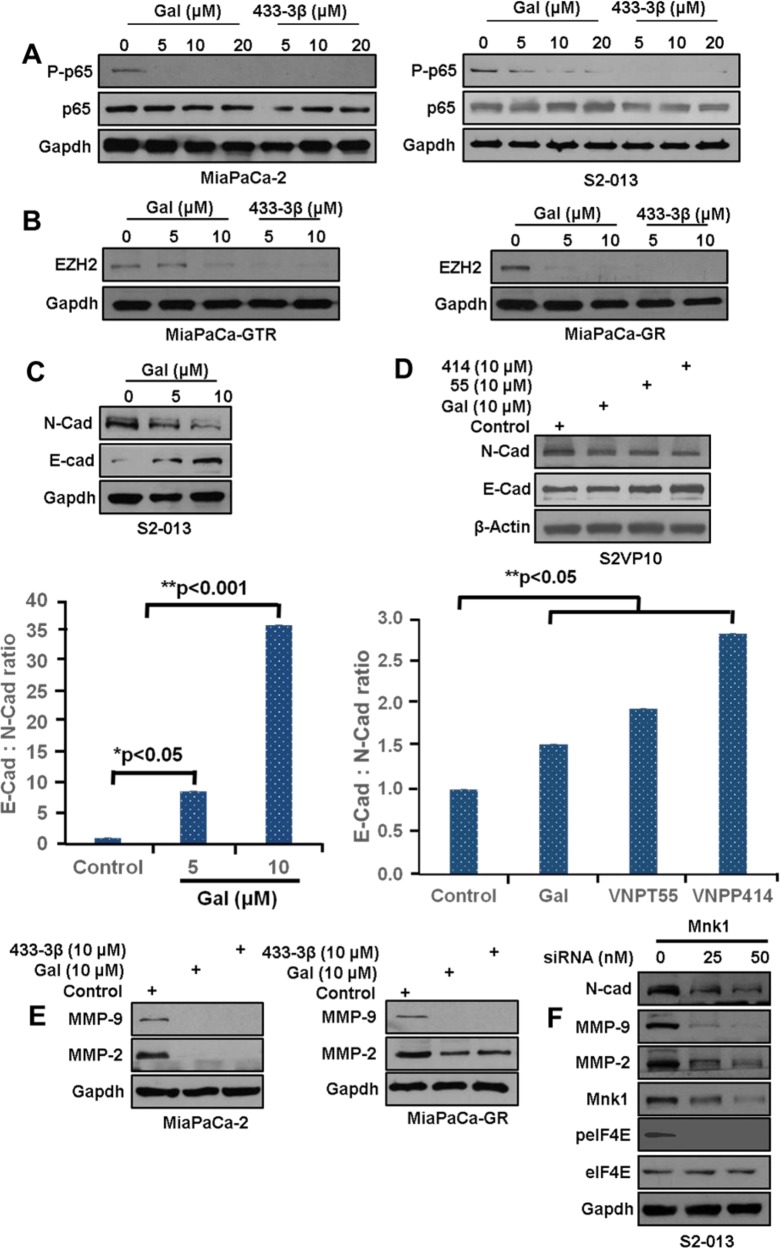
Gal and analogs inhibit metastatic inducing pathways **A**. MiaPaCa-2 and S2-013 cells treated with gal and VNPP433-3β at increasing concentration show a dose-dependent decrease in p-p65. **B**. MiaPaCa-GR and MiaPaCa-GTR cells exposed to gal and VNPTP433-3β show profound depletion of EZH2 protein expression MiaPaCa-GTR (*left panel*) and MiaPaCa-GR (right *panel*). **C**. S2-013 cells, left *panel*, exposed to gal (5 – 10 μM) show downregulation of N-cadherin, and an upregulation of E-cadherin (*bottom graph*). Densitometric analysis suggests a profound increase in E-Cad: N-Cad ratio, suggesting loss of mesenchymal characteristics. **D**. S2-VP10 cells exposed to 10 μM of gal/analogs exhibit a downregulation of N-cadherin and an upregulation of E-cadherin resulting in an increase in E-cad N-cad ratio (*bottom chart*). **E**. Gal and VNPP433-3β deplete MMP-2/-9 in MiaPaCa-2 and MiaPaCa-GTR cells after 24 h treatment period. **F**. S2-013 cells transfected with Mnk1 siRNA show downregulation of N-cadherin, MMP-2/-9 and peIF4E.

The NF-κB pathway, implicated in cancer cell migration and invasion is also involved in gemcitabine-resistance in PDAC [17, 18, 35]. Gal and VNPP433-3β caused a marked inhibition of NF-κB (p65) phosphorylation (p-p65) in both MiaPaCa-2 and S2-013 cells (Figure 4A, *left and right panels*).

Enhancer of Zeste 2 Polycomb Repressive complex subunit 2 (EZH2), which has been shown to induce resistance and cancer cell migration and invasion was markedly down-regulated in MiaPaCa-GTR and MiaPaCa-GR cells (Figure 4B, *left and right panels respectively*). EZH2 is reported to be highly expressed in pancreatic cancer cells, known to silence E-Cadherin and it has also been implicated in MMPs activation [36]. We next examined the effects of gal on N-cadherin and E-cadherin expression in the metastatic cell lines S2-013 and S2-VP10). In S2-013 metastatic cells, gal, dose-dependently, decreased N-cadherin and increased E-cadherin protein expression (Figure 4C, resulting in a strikingly high E-cadherin: N-cadherin ratio, suggesting a possible reversal in EMT activity (Figure 4C, *bottom graph*). To determine whether analogs of gal exhibited similar characteristic on E/N-Cadherin, S2-VP10 cells, were also treated with gal, VNPT55 and VNPP414. Protein expression analysis show similar results with a significant increase in E-Cadherin: N-cadherin ratio (Figure 4D, *top and bottom panel)*.

MMP-9, one of the two type IV collagenases, essential in degrading extracellular matrix during invasion is reported to be overexpressed in pancreatic cancer [37–39]. Gal and VNPP433-3β strongly depleted MMP-2/-9 in MiapaCa-2 and MiaPaCa-GR PDAC cells (Figure 4E, *left and right panel*). Several lines of evidence have shown that knocking down Mnk1 resulted in a downregulation of eIF4E phosphorylation with a significant negative impact on EMT and stem cell markers. Our complementary, siRNA knockdown of Mnk1 in S2-013 cells also caused marked down-regulation of N-Cadherin, MMP-2/-9 and eIF4E phosphorylation (Figure 4F). This suggests that, by depleting Mnk1/2, gal and its analogs modulate these proteins, in part, via the Mnk-eIF4E pathway. A study by Soifer *et al*., showed that gal treatment dose-dependently decreased interaction between eIF4G and eIF4E, using the 7mG pulldown assay [40]

**Figure 5:**
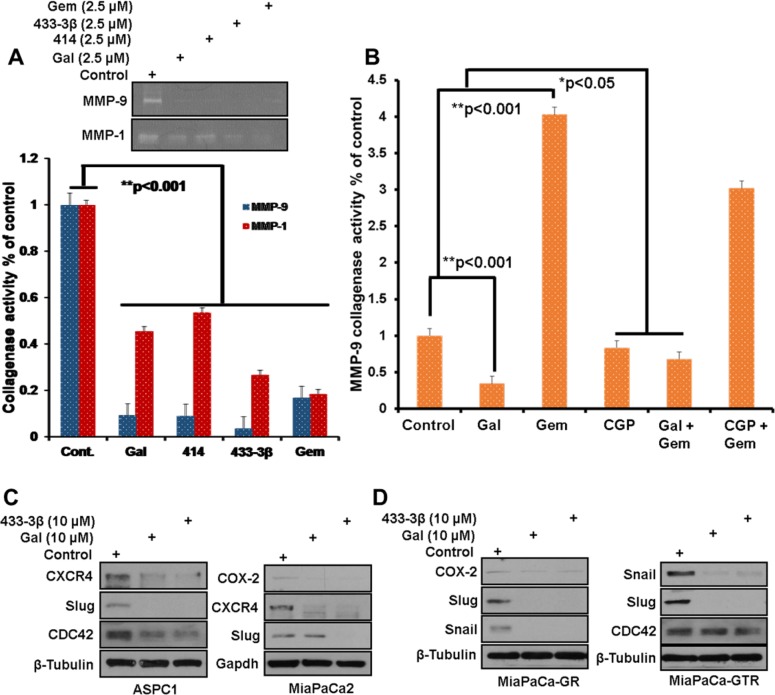
Downregulation of EMT markers after **A**, *top panel*) Media from S2-013 cells, treated with indicated compounds was separated on a zymogram gel to analyze the proteolytic activity of MMP-1/-9. (**A**, *bottom graph*) densitometric analysis of zymogram gel in *top panel*, reveals a significant decrease in collagenase activity of MMP-1/-9 (**p < 0.001), zymogram assays were repeated at least three times and presented as mean ± S.E.M. **B**. Densitometry of proteolytically digested bands on zymogram gels analyzed from gal, CGP, gemcitabine and combinations’ treated MiaPaCa-2 cultures, shows significant upregulation of MMP-9 collagenase activity in the presence of gemcitabine. Gal and CGP both decrease gemcitabine-induced MMP-9 activity (*p < 0.05, **p < 0.001). **C** and **D**. Gemcitabine-naïve, Gemcitabine-resistant and Gemcitabine/erlotinib resistant, PDAC cells, were treated with gal and VNPP433-3β for 24 h at 10 μM. Protein expression analysis show downregulation of Snail, Slug, Cox-2, CDC42 and CXCR4 in all four cell lines.

Zymogram analysis of MMP-1/-9 collagenase activity secreted by S2-013 cells interestingly showed that both ARDAs and gemcitabine significantly decreased MMP-1/-9 collagenase activity (Figure 5A, *top gel and bottom graph*). However, in MiaPaCa-2 cells, treatment with gemcitabine alone increased secreted MMP-9 collagenase activity and co-treatment with gal or CGP-57380 significantly suppressed gemcitabine-induced MMP-9 collagenase activity (Figure 5B). EMT markers are implicated in disease progression of a number of malignancies including pancreatic cancer [41–43]. During cell migration, activated EMT markers, such as CDC42 (cell division cycle 42, a GTP-binding protein) are involved in cell cytoskeletal reorganization, cell polarity, cell motility and invasion [44, 45]. In this study, we observed significant down-regulation of CDC42 protein expression in ASPC1 and MiaPaCa-GTR cells (Figure 5C and 5D), which could represent a possible molecular mechanism for gal/VNPP433-3β suppression of PDAC cell migration.

Interestingly, protein expression analysis after PDAC cells were exposed to gal or its analogs (10 μM), showed striking decreases in Snail, Slug and COX-2 (Figure 5C and 5D). We also observed that gal and its analogs profoundly depleted protein expression of CXCR4 (Figure 5C and 5D, *ASPC1 and MiaPaCa-2*).

### Gal and analogs inhibit gemcitabine-naïve/resistant PDAC cell migration and invasion

**Figure 6:**
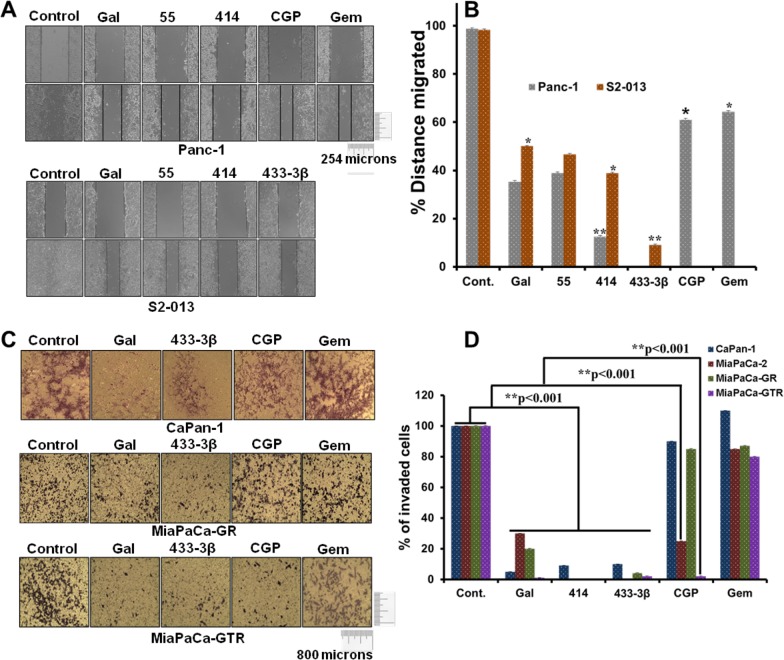
Gal and analogs possess anti-migratory and anti-invasive properties **A**. Scratch wound healing assays reveal that gal/analogs, CGP and gemcitabine at 5 μM, inhibit Panc-1(*top panel*) and S2-013 (*bottom panel*) PDAC cell migration. **B**. Distance migrated by Panc-1 and S2-013 cells after treatment were quantified. Wounds were measured before and after the 12 h time point. Distance migrated were quantified by measuring the difference at time 0 and 12 h and normalized to control. (Distance migrated = Distance at time 0 hour - distance at 12 h/ Distance migrated by control) (*p < 0.05, **p < 0.001). **C**. Gal/analogs, CGP, gemcitabine at 5 μM, inhibit gemcitabine-naive (CaPan-1), MiaPaCa-GR and MiaPaCa-GTR PDAC cell invasion. **D**. Quantification of invaded PDAC cells was done by counting cells in quadrants. Only invading cells at the bottom of inserts were counted. Quantified invaded cells shows a significant inhibition of PDAC cell invasion (**p < 0.001).

Following the significant depletion effects observed on EMT markers, the next logical step was to evaluate and validate potential anti-migratory and anti-invasive properties of gal and its analogs in PDAC cell lines *in vitro* with functional assays. Migration assays were performed over a 12 h time point, whereas invasion assays were incubated for 24 h. At 5 μM, gal and its analogs inhibited migration of Panc-1 and S2-013 PDAC cells significantly compared to DMSO treated controls (Figure 6A and 6B). We also observed that both gemcitabine and CGP-57380 decreased migration of Panc-1 cells. Migration assays showed that VNPP414 and VNPP433-3β were more potent in inhibiting PDAC cell migration than gemcitabine and CGP-57380 in gem-naïve cells (Figure 6A and 6B).

Although Panc-1 cells have been shown to be more resistant to gemcitabine [46], *in vitro* anti-migratory assays revealed significant activity of gemcitabine in gemcitabine-naïve Panc-1 cells. However, gemcitabine lacked significant anti-invasive properties, both in gemcitabine-naïve and gemcitabine-resistant PDAC cells (Figure 6C and 6D), contrary to the effects seen with gal and its analogs. Interestingly, CGP-57380 which previously has been shown to inhibit eIF4E phosphorylation, also exhibited significant inhibition of MiaPaCa-2 and MiaPaCa-GTR cell invasion (Figure 6C and 6D). This clearly suggests that the Mnk-eIF4E pathway plays a very significant role in invasion of gemcitabine/erlotinib-resistant PDAC cells.

### Gal and its analogs deplete putative stem cell factors and reduce PDAC clonogenicity and spheroid formation

**Figure 7:**
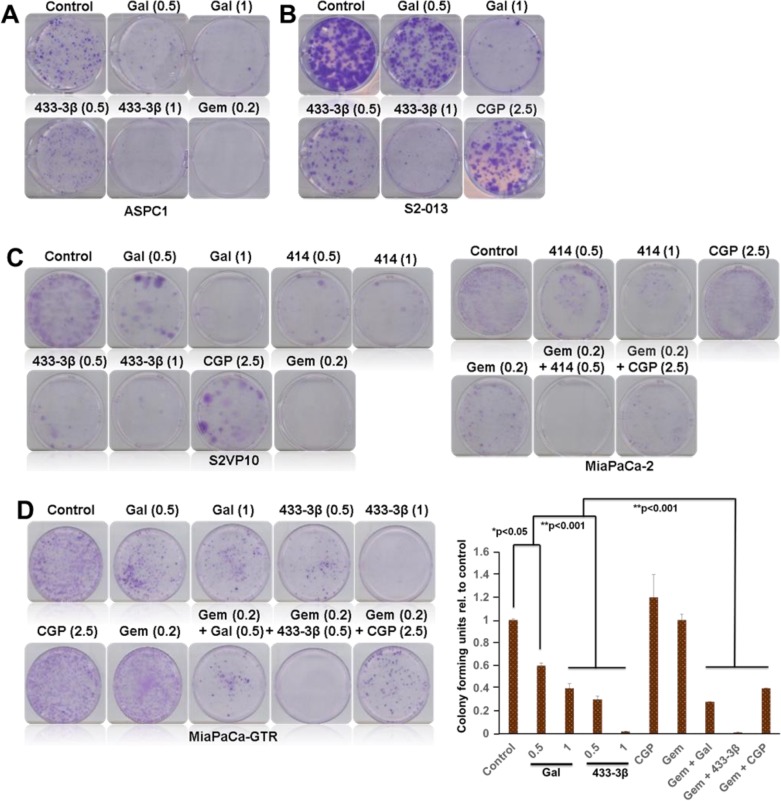
Gal and analogs inhibit colony formation of PDAC cell **A**. Gal/VNPP433-3β and gemcitabine significantly inhibit colony formation of ASPC1 and **B**. S2-013 cells. **C**. Gal/VNPP414/VNPP433-3β/CGP and gemcitabine decreased colony formation of S2-VP10 (*left panel*) and MiaPaCa-2 (*right panel*) cells. Both VNPP414 and CGP potentiated the effects of Gemcitabine and severely reduced colonies (**C**, *right panel*) in MiaPaCa-2 cells. **D**. Gal and VNPP433-3β significantly inhibited colony forming units (CFU) of gemcitabine-erlotinib-resistant PDAC cells and potentiated the effects of gemcitabine in further decreasing the number of colonies formed, contrary to gemcitabine alone which did not show strong inhibition of colonies. (**D**, *right graph*) Colony formation assays were repeated at least three times and colonies counted in four quadrants of the wells. Results are represented as averages with S.E.M. (*p < 0.05, **p < 0.001). Note: The numbers in parenthesis are concentrations in μM.

Gal and analogs have displayed consistent activity in inhibiting proliferation of gemcitabine-naïve and gemcitabine-resistant PDAC cells. Colony formation assays were performed with 1000 cells seeded in 6-well plates. After cells attached, they were subsequently treated with ARDAs alone or in combination with gemcitabine or CGP-57380. Results from cell types ASPC1 (Figure 7A) and S2-013 (Figure 7B), after a 14-day treatment period show strong inhibition of colony formation. Gal and analogs exhibited similar activity in gemcitabine-naïve S2-VP10 cells (Figure 7C, *left panel*). Gemcitabine treatment in MiaPaCa-2 was not as efficacious as in ASPC1 and S2-VP10 (*compare* Figure 7A and 7C). However, in combination with VNPP414, effects were potentiated in inhibiting formation of colonies. In gemcitabine/erlotinib-resistant cells, both gal and VNPP433-3β profoundly decreased colony formation (Figure 7D, *left and right panels*), effects of which were highly potentiated in combination with gemcitabine (Figure 7D). In addition, combining VNPP414 or CGP-57380 with gemcitabine showed enhanced activity compared to the single agents (Figure 7D, *right panel*). Relative to controls, colonies resulting from gemcitabine-CGP-57380 combination were significantly inhibited, contrary to considerably insignificant effects with either single agent (Figure 7D), emphasizing the potential significance of Mnk inhibition in pancreatic cancer therapy.

**Figure 8:**
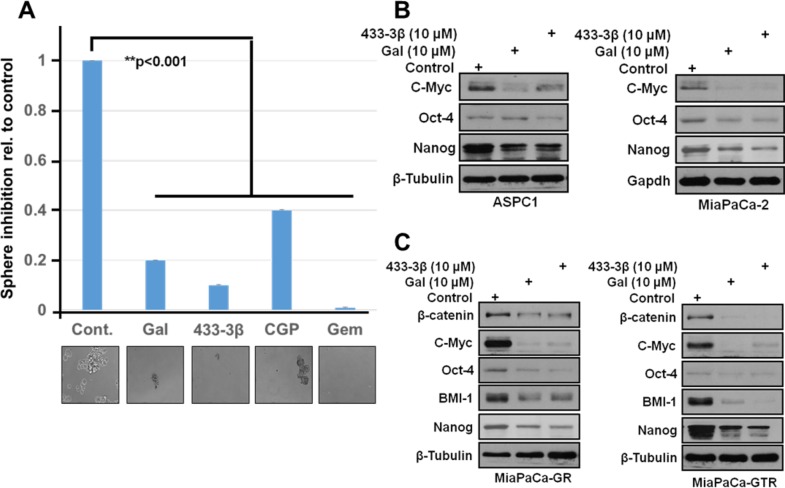
Gal and analogs downregulate stem cell factors and inhibit sphere formation in S2-013 PDAC cells **A**. 200 cells resuspended in media to break up clumps and ensure single cell suspension were seeded in ultra-low adherent 24-well plates. After sphere formation, cells were treated with indicated compounds 2X in 14days and spheroids analyzed by light microscopy and images taken. **B** and **C**. Gal and VNPP433-3β deplete protein expression of putative stem cell factors in gemcitabine-naïve and gemcitabine-resistant PDAC cells. β-Catenin, Oct-4, Nanog, BMI-1 and cell proliferation factor c-Myc were downregulated after 24 h treatment period.

Recent studies on cancer stem cells and EMT-type cells revealed the crucial role they play in drug-resistance and metastases [47]. The spheroid formation assay was adapted from previously published work [48, 49]. 200 cells were seeded in a ultra-low adherent 24-well plate with sphere forming media (1:1 DMEM/F12 medium supplemented with B-27 and N-2; Invitrogen). Cells were treated with 2.5 μM of the indicated compounds for 14 days, media and compounds were replenished once. Sphere formation was analyzed by counting spheres formed and images taken. Representative images are as shown in Figure 8A. Results show that gal, VNPP433-3β and gemcitabine all significantly decreased formation of S2-013 spheroids. These results mirror what was observed in our colony formation assay. Protein expression analysis of gal/analogs-treated gemcitabine-naïve (ASPC1 and MiaPaCa-2) and gemcitabine-resistant (MiaPaCa-GR and MiaPaCa-GTR) PDAC cells show a marked down-regulation of putative stem cell factors (Nanog, BMI-1, β-Catenin and Oct-4) (Figure 8B and 8C). Immunoblot analysis revealed that gal/analogs potently decreased protein expression of c-Myc in ASPC1, MiapaCa-2, MiaPaCa-GR and MiaPaCa-GTR cells (Figure 8B and 8C). This effect may contribute to the anti-proliferative activities of these compounds.

### Gal, VNPT55, VNPP414 and VNPP433-3β suppress PDAC MiaPaCa-2 xenografts in mice

**Figure 9:**
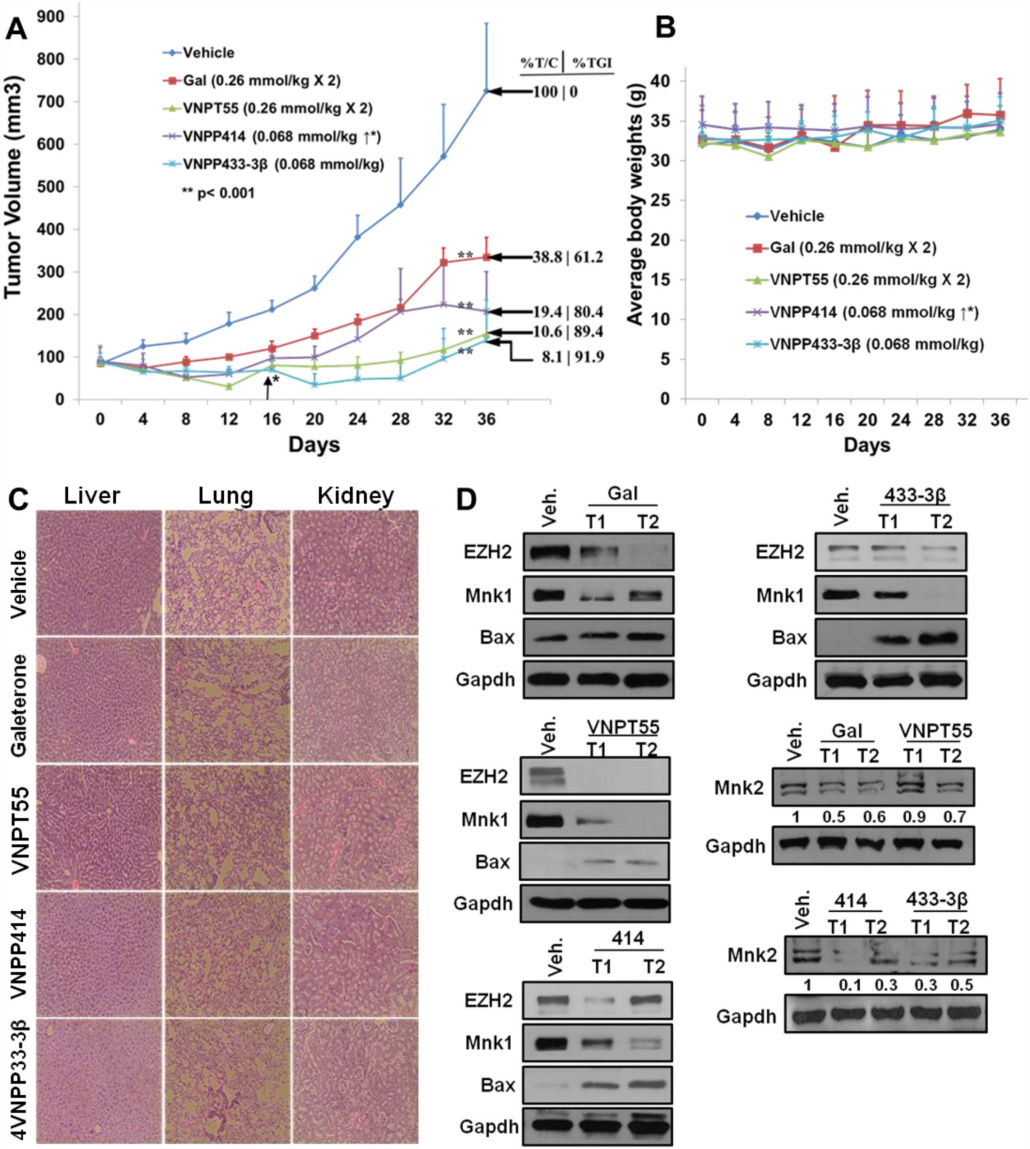
*In vivo* activity of gal and analogs **A**. Effect of gal, VNPT55, VNPP414 and VNPP433-3β were evaluated in MiaPaCa-2 PDAC xenograft-bearing mice. Mice (n = 5) were administered with gal [0.26 mmol/kg (100.9 mg/kg)/twice daily], VNPT55 [0.26 mmol/kg (125.4 mg/kg)/twice daily], VNPP414 [0.068 mmol/kg (32.6 mg/kg)/twice daily], and VNPP433-3β [0.068 mmol/kg (30 mg/kg)/once daily], by intraperitoneal injection, 5 days per week for 32 days. Tumors were measured twice a week. Gal and analogs significantly inhibited tumor growth (** p< 0.001; %T/C: ratio of tumor volume in treated mice *vs*. control). Effective criteria for %T/C value according to NCI standard is ≤42% [50, 51]. TGI: Tumor Growth Inhibition index = [1-(mean volume of treated tumors)/(mean volume of control tumors)] × 100%). **B**. Mean body weights of mice were taken twice a week for the duration of the study. Mean body weights showed no significant toxicities to mice. **C**. H & E stain of liver, lung and kidney in both compound treated animals and vehicle treated animal show no gross organ abnormalities. **D**. Western blot analyses show a marked depletion of Mnk1/2, EZH2 and an upregulation of proapoptotic protein Bax by all four compounds *in vivo*.

To demonstrate the anti-PDAC activity *in vivo*, we developed PDAC tumor xenografts from MiaPaCa-2 cells and subsequently treated with intraperitoneal (i.p.) administration of gal or the analogs when tumors reached ∼85 mm^3^ in size. Two groups were treated with gal or VNPT55, (0.26 mmol/kg, twice daily, 5 days/week), a third group received VNPP414 (0.068 mmol/kg, twice daily, 5 days/week), whilst the VNPP433-3β group received 0.068 mmol/kg once daily, 5 days/week for 36 days as described in Materials and Methods. The dose selections was based on our previous studies with gal and VNPT55 [27] and on the projected efficacies of VNPP414 and VNPP433-3β. As shown in Figure 9A, VNPP433-3β was the most effective therapeutic agent in inhibiting the growth of MiaPaCa-2 tumors. The decreasing order of potency (tumor growth inhibition, TGI) was: VNPP433-3β (91.9%, *p* < 0.001 *vs*. control) > VNPT55 (89.4%, *p* < 0.001 *vs*. control) > VNPP414 (80.4%, *p* < 0.001 *vs*. control) > gal (61.2%, *p* < 0.001 *vs*. control). The tumor growth inhibition, measured as %T/C, ranging from 8.1 to 38.8% (Figure 9A), classify these agents as highly efficacious according to the National Cancer Institute's (NCI’s) criteria [50, 51]. Tumor growth inhibition (%T/C), is defined as the ratio of the median tumor volume for the treated versus control group. Of particular interest, we found that the average tumor sizes were lower than the initial sizes (∼85 mm^3^) in mice in the VNPT55 and VNPP433-3β treated groups after 16 and 28 days’ treatments, respectively, suggesting that these treatments caused partial tumor regressions (Figure 9A). In general, no host toxicity was observed, since there were no significant differences in the body weights between the control group and the mice treated with gal or its analogs (Figure 9B). However, in the VNPP433-3β treated group, two mice were found dead on day 28 with no preceding weight loss or apparent cause. Additionally, the H & E staining of liver, lung and kidney in the vehicle-treated and agent-treated groups, at day 36 did not show any gross organ abnormalities following histological examinations. (Figure 9C). Protein expression analysis by western blotting also confirmed effects observed *in vitro*, where gal and analogs depleted Mnk1/2, EZH2 and up-regulated Bax expression, suggesting a possible apoptotic induction *in vivo* (Figure 9D).

**Figure 10:**
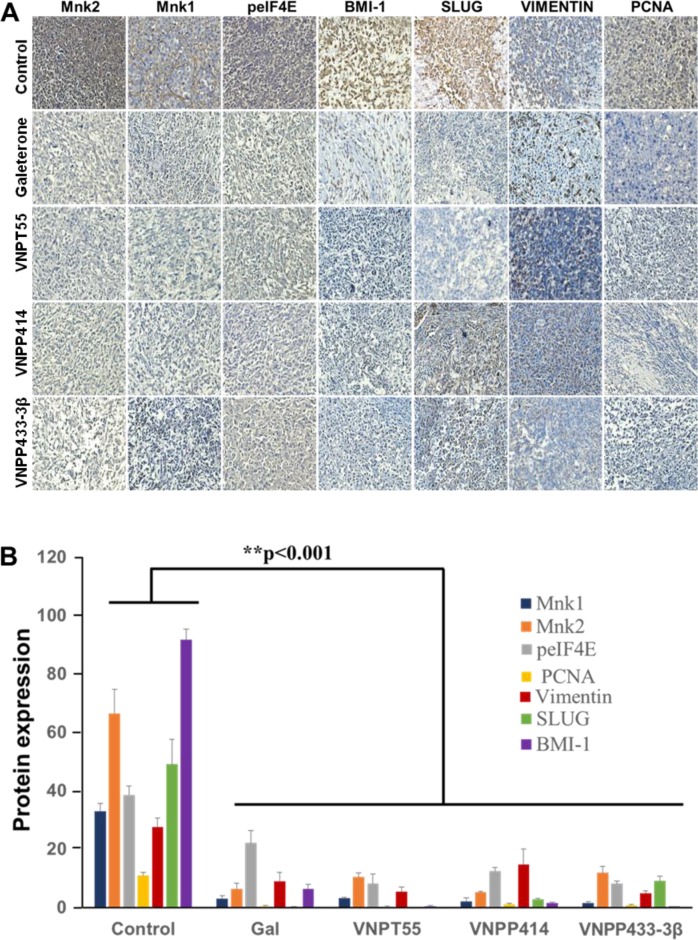
Downregulation of oncogenic biomarkers *in vivo* **A**. Immunohistochemical staining show significant depletion of Mnk1/2, peIF4E, BMI-1, SLUG, vimentin and PCNA from paraffinized sections of tumor tissue. **B**. Quantified IHC stains, show a significant downregulation of biomarkers *in vivo* (**p < 0.001).

To further validate the *in vitro* findings regarding the effects of our compounds on key oncogenic biomarkers, immunohistochemistry (IHC) analysis was conducted to quantitate the expression of suppressed Mnk1/2, BMI-1, Slug and peIF4E. Consistent with the *in vitro* data, all four compounds caused profound depletion of the key components of protein translation machinery, including, Mnk1, Mnk2, peIF4E, in addition to significant decreases in BMI-1, vimentin and Slug (Figure 10A). Furthermore, PCNA expression was also down-regulated *in vivo*, suggesting that gal and its analogs inhibit also cell proliferation *in vivo* (Figure 10B).

## DISCUSSION

Multi-targeted single-agents and drug combinations are spearheading pancreatic cancer therapeutics to minimize resistance and enhance efficacy [46]. By targeting and inhibiting multiple oncogenic pathways simultaneously, drug combinations potentially may offer a major advantage over “single-target” therapy [52]. Gemcitabine, gemcitabine/erlotinib and Folfirinox are the respective elective single-agent and drug combinations in PDAC chemotherapy. These drugs exert only marginal survival benefits with increased patient toxicity, emphasizing the urgency to identify new non-toxic therapeutic agents that offer superior activity against resistance-inducing oncogenes responsible for PDAC drug resistance.

Recent studies have shown that gal and analogs have the unique ability that effectively modulate oncogenic eukaryotic protein translation via depletion of Mnk1/2 and downregulation of eIF4E phosphorylation [24]. NF-κB activation is associated with the down-regulation of nucleoside transporters (hCNT1) in gemcitabine-naïve PDAC, which results in significant decrease in gemcitabine uptake [53], hence the inefficacy reported in gemcitabine therapy. Thus, combining gal or its analogs with gemcitabine has the potential of increasing gemcitabine uptake by cells, thereby potentiating its anti-tumor activities.

By downregulating NF-κB phosphorylation, these compounds may also inhibit NF-κB activation of target genes, which may result in inhibiting the metastatic potential of PDAC cells. In acquired PDAC drug resistance, these unique mechanisms of gal and its improved analogs may offer an advantage over currently available drugs for PDAC therapy. The *in vitro* results from this study show that gal and its analogs exhibit profound anti-PDAC activity by inhibiting cell proliferation, colony formation, spheroid formation, cellular migration and invasion in addition to apoptotic induction. These novel small molecules potentiated the effects of gemcitabine and synergistically enhanced the efficacy of gemcitabine in drug-resistant cells with very low CI values ranging from 0.03-0.4. These effects may be associated with inhibition of Mnk1/2-eIF4E axis and EMT markers (N-cadherin, Snail, Slug and EZH2) in addition to other activities, which were not investigated. Induction of apoptosis in cells isolated from primary tumors, metastatic lesions and gemcitabine resistant cells, indicate their potential in targeting all forms and stages of PDAC. K-RAS oncogenic activation has been implicated in EZH2 up-regulation in pancreatic cancer [54].

Interaction between cancer cell-stroma is implicated in the high metastatic potential characteristic of PDAC [55]. Several reports have emphasized the significance and pivotal role matrix metalloproteinases (MMPs) play in cancer cell invasion and metastasis [56], which interestingly is up-regulated in invading cancer cells. Cyclooxygenase-2 (COX-2), implicated in cell invasion is also overexpressed in PDAC [57–59], and studies have shown a strong positive correlation between MMP-9 and COX-2 expression [60] in PDAC. Gal and VNPP433-3β's inhibitory effect against Mnk2, COX-2 expression and secreted MMP-9, suggests the potential anti-invasive activity of these agents. Interestingly, gemcitabine-induced collagenase activity of MMP-9 was significantly suppressed when combined with gal in gemcitabine-resistant cells. Recent reports have emphasized the significance of MMP-1 expression in pancreatic cancer and its correlation with poor patient prognosis [61]. Thus, the effects of these compounds on MMP-1 activity may play a significant role in potential inhibition of PDAC disease progression in patients

Several other EMT markers such as Snail, Slug and Twist 1 have been implicated in increased tumor grade of pancreatic cancer [62]. Snail has also been reported to correlate with lymph node and distant metastases [63] and increased fibrosis *in vivo* [64]. β-Catenin and CXCR4 have also been implicated in pancreatic cancer progression and metastasis and CXCR4 expression has been correlated with poor survival in PDAC patients [65]. Down-regulating these factors, potentially can contribute to the efficacy of these novel inhibitors in PDAC therapy, as observed *in vitro* and *in vivo*. In this study, there is evidence of the efficacy of our compounds as anti-PDAC therapeutic agents. Deregulation of c-Myc has been reported to be common in early stages of pancreatic cancer disease and its progression [66]. A recent study also showed that gli2-induced overexpression of c-Myc is implicated in pancreatic cancer cell resistance to JQ1 and 1-BET151, selective inhibitors of BET bromodomain proteins [67]. These compounds exhibit diverse activities in inhibiting several biological functions and molecular pathways employed by PDAC to increase morbidity.

Perhaps the most significant piece of data resulting from this study, is the activity that these compounds exhibited *in vivo*. Xenograft tumors developed from MiaPaCa-2 cells, which exhibit strong innate resistance to gemcitabine, were significantly and strongly inhibited. The tumor growth inhibitions (TGIs) relative to vehicle control at the end of the study (36 days), were very impressive, ranging from 61% to 92% (*p* values < 0.001), emphasizing the significance of the anti-tumor efficacies of these agents in a difficult-to-treat PDAC xenograft model. Of additional interest and significance, is the finding that VNPP433-3β was far more efficacious against MiaPaCa-2 tumor xenografts than a 7.65-fold higher dose of gal. Impressively too, was the observation that Mnk1, Mnk2, peIF4E, PCNA, EZH2 and Bax were all positively modulated *in vivo*, indicating the reproducibility of the compounds activities, both *in vitro* and *in vivo*, thereby, validating their mechanisms of action.

In summary, we have demonstrated that gal and its new analogs are potent inhibitors of pancreatic cancer cell growth and, they also induce strong cell cycle arrest and apoptosis. The agents also inhibit gemcitabine-resistant PDAC cell growth and exhibit synergistic effects with gemcitabine. We also establish that gal and its analogs are potent inhibitors of the growth of PDAC cells derived from primary localized tumors and metastatic lesions. We also documented attenuation of several oncogenic signaling pathways, including, Mnk1/2, peIF4E and NF-κB (p65) phosphorylation, metastatic inducing factors (N-cadherin, MMP-1/-2/-9, Slug, Snail and CXCR4) and putative stem cell factors, (β-Catenin, Nanog, BMI-1 and Oct-4). These impressive anti-PDAC activities together with their strong anti-migratory and anti-invasive activities, *in vitro* and strong inhibition of MiaPaCa-2 tumor growth suggest that gal and its analogs have great potential either as monotherapies or in combination with current elective PDAC drugs for the treatment of various forms of pancreatic cancer. Given the potential that well-tolerated gal would soon be approved for the treatment of prostate cancer patients, drug repositioning for PDAC therapy would limit the costs and reduce risk. In addition, because of the remarkable superiority of VNPP433-3β over gal with respect to their anti-tumor efficacies, VNPP433-3β could advance rapidly through Investigational New Drug (IND)-enabling studies, in view of Phase 1 clinical trials in men and women with pancreatic cancer.

## MATERIALS AND METHODS

### Cell culture

Human PDAC cells lines (Panc-1, HS766T, MiaPaCa-2, S2-013, S2VP10, ASPC1 and CaPan1) were purchased from ATCC and maintained in DMEM supplemented with 10% fetal bovine serum, 1% penicillin-streptomycin (penstrep) and 5% L-Glutamine. MiaPaCa-2 cell obtained from ATCC were made resistant to gemcitabine: MiaPaCa-GR (200 nM of gemcitabine) and MiaPaCa-GTR (2 μM of erlotinib and 200 nM of gemcitabine). These drug-resistant cell lines were acquired from Dr. Fazlul H. Sarkar, Wayne State University School of Medicine, Detroit, Michigan; and maintained in DMEM as in the gemcitabine-naïve cells.

### Reagents, chemicals and antibodies

Galeterone and analogs (VNPT55, VNPP414 and VNPP433-3β) were designed and synthesized in our laboratory [26, 68, 69] and dissolved in DMSO. Gemcitabine was purchased from Sigma Aldrich. CGP-57380 was purchased from Eli Lilly. Cell culture reagents (FBS, RPMI, and DMEM) were from Invitrogen. β-catenin, Cox-2, Oct-4, Nanog, β-actin, β-Tubulin, Gapdh, Mnk1/2, eIF4E, peIF4E, N-cadherin, E-cadherin, Snail, Slug, MMP-2/-9, BMI-1, NF-κB (p65, p52), caspase 3, PARP anti-mouse and anti-rabbit HRP were purchased from cell signaling. C-Myc, Bax, Bcl-2, K-RAS, CXCR4, cyclin B1, cyclin D1, CDC42 and EZH2 antibodies were purchased from Santa Cruz biotech.

### Immunoblot

PDAC cells synchronized in serum free media were maintained and treated in regular media with varying concentrations over indicated time points. RIPA lysis buffer (Sigma Aldrich), supplemented with 1X protease inhibitors (protease cocktail from Roche), and 1 mM EDTA were used in cell lysis. Immunoblot analysis was performed as previously reported [27].

### Cell viability assays (MTT 3-(4, 5-dimethylthiazol-2-yl)-2, 5-diphenyltetrazolium bromide, colorimetric assay)

MTT cell viability assays were performed as described in our previous publications [26]. Briefly, 2000-3000 cells were seeded in 96-well plates overnight to allow cells to attach. Cells were subsequently treated with indicated compounds for the duration of 7 days.

### Combination studies

Compounds used in drug combination were added simultaneously at their respective GI_50_ values, maintaining a fixed ratio. MTT cell viability assay was utilized to determine fraction of cells affected (FA). Combination index (CI) to evaluate synergy, additivity or antagonism was computed using the calcusyn software (Biosoft, Ferguson, MO and Cambridge, UK), following Chou-Talalay method [31]. Combination was synergistic: CI<1, additive: CI=1 and antagonistic: CI>1.

### Cell cycle analysis

Cells were seeded in 6-well plates at 60% confluence overnight to allow cells to attach. The cells were then subsequently serum starved with phenol-red free RPMI to arrest the cells at G0/G1 phase. After 12 h, serum-free media was replaced with complete DMEM media supplemented with 10% FBS and cells treated with compounds at (5-20 μM) for 24 h. After the indicated time point, cells were washed 2X with PBS and thoroughly resuspended in 200 μl of phosphate-buffered saline (PBS). Ethanol was then added for a final ethanol percentage of 80% (fixation). The fixation step was incubated at 4°C overnight. Cells were washed twice with 2 ml PBS and pelleted at 800g for 5 minutes and resuspended in 500 μl Propidium Iodide staining buffer.

### Apoptosis assay

The Acridine orange (AO) and ethidium bromide (EB) (Sigma Aldrich) apoptotic detection assay was used to determine apoptotic cells in S2-013 cells. Cells were treated in 6-well plates at 2.5 μM. After 72 h cells were washed 1X with warm PBS and incubated in 400 μl of 0.1% EB and 0.2% AO in PBS at 37°C for 30 minutes. AO/EB buffer was washed off 1X with warm PBS and images immediately taken using fluorescence microscope Nikon TE2000 microscope

FACS analysis was performed on compound treated MiaPaCa-GR cells to determine apoptosis induction. Cells were exposed to 5 and 10 μM of galeterone/analogs and gemcitabine. After 24 h, annexin-V-FITC apoptosis detection kit (BD Biosciences) was used following manufacturers protocol.

### SiRNA transfection

S2-013 cells were transfected with 25 and 50 nM Mnk1 siRNA (Invitrogen) for 72 h. Scrambled siRNA were transfected in the control wells. 20 μl of lipofectamine RNAiMAX (Invitrogen) reagent was incubated with siRNA in 1 ml of OPTI-MEM media in Eppendorf tubes for 15 minutes (room temperature, *r.t*.). 100 mm plates were subsequently coated with siRNA complexes for an additional 15 minutes. 3 ml of cell suspended in DMEM media without pen/strep were added to plates for 16 h, Cells were lysed after 72 h and protein expression analyzed.

### Analysis of secreted MMP-1/-9 collagenase activity

Cells were treated with ARDAs or gemcitabine in phenol red free media, with no penicillin/streptomycin for 72 h. Cultured media from was collected and concentrated with 0.5 ml amicon ultra centrifugal columns (Millipore, Bedford, MA). Media containing secreted protein was quantified and separated on a 0.1% gelatin SDS-polyacrylamide gel. Gels were incubated in 1X renaturing buffer for 20 minutes (2X), at r.t. Gels were incubated in 1X developing buffer for 48 h and stained with page blue. Bands were developed with de-staining solution (50% methanol, 10% acetic acid and 40% water).

### Cell motility (scratch-wound-healing) assay

5 × 10^5^ cells (Panc-1 and S2-013) were cultured in 24-well plates and allowed to form a uniform monolayer. Cells were maintained in serum-free media for additional 12 h and subsequently scratched with a 200 μl pipette tip. Cells treated with indicated compounds and concentrations were incubated for 12 h at 37°C in regular media. Images and subsequently taken (at 0 and 12 h) using the Zeiss microscope and distance migrated measured.

### Invasion assay

Trevigen basement membrane extract (BME) pre-coated transwell inserts were placed in 37°C incubator for 2 h to allow inserts to attain the requisite temperature. 1 × 10^4^ cells were seeded in the top chamber in serum-free media with or without indicated concentrations of compounds. The bottom chamber was filled with 1 ml of regular media with 10% fetal bovine serum to serve as chemoattractant. The set-up was placed in a 37°C incubator. After 24 h, cells remaining at the top chamber were scraped off with cotton swabs and migrated cells at the bottom of the inserts were fixed with ice cold methanol and stained in 0.05% crystal violet.

### Colony formation assay

0.5 × 10^3^ cells seeded in 6-well plates were allowed to attach overnight, and were subsequently treated with compounds in regular at the indicated concentrations. Media containing compounds were replaced every 3^rd^ day for 14 days. Colonies were washed 1X with PBS and stained with 0.05% crystal violet for 30 minutes. Scanned colonies were quantified with ImageJ colony counter. All experiments were repeated at least 3 times and represented as means ± S.E.M.

### Sphere formation assay

To evaluate the ability of gal and its analogs to inhibit pancreatic cancer stem cells renewal, we adapted the protocol from Sarkar *et al*. [48] and Majumdar *et al*. [49]. Briefly single cell suspension was seeded in ultra-low adherent 24 well plates. 200 cells per well were seeded the suspended in serum-free stem cell medium containing DMEM/F12 (1:1) supplemented with B27 and N-2 (Life Technologies, Gaithersburg, MD). Cells were incubated at 37°C for 10 days until pancreatospheres formed. Spheres were then incubated with or without galeterone, VNPP433-3β, CGP-57380 and gemcitabine at 2.5 μM for 14 days. Media was replenished 1X and the number of pancreatoshperes formed were evaluated by light microscopy.

### Xenograft study

All animal studies were performed as previously reported [27]. Male severe combined immunodeficiency (SCID) mice 5-6 weeks of age were obtained from the National Cancer Institute (Fredrick, MD) Approximately 2×10^6^ MiaPaCa-2 cells were inoculated into both flanks of mice. When tumor-reached approximately 85 mm^3^, mice were randomized into 5 groups of 5 mice per each group. Compounds were formulated in 40% β-cyclodextrin in ddH2O and administered intraperitoneal. Tumors measurements were taken twice weekly and tumor volume was computed using the formula: length × width^2^ × 0.5 (mm^3^). Animal weights were also taken twice weekly, monitoring the general health and activity of animals. Mice were euthanized at the end of the study and tumors and organs excised. Tumors sections were taken and frozen or retained in 10% buffered formalin for further analysis.

### Statistical analysis

All experiments are reported as means with standard error where applicable. Student T-test and Analysis of variance (ANOVA) were utilized to perform statistical analysis and where applicable, to determine the significance of observed results.
